# Scalable and controlled self-assembly of aluminum-based random plasmonic metasurfaces

**DOI:** 10.1038/lsa.2017.15

**Published:** 2017-07-14

**Authors:** Radwanul Hasan Siddique, Jan Mertens, Hendrik Hölscher, Silvia Vignolini

**Affiliations:** 1 Institute for Microstructure Technology, Karlsruhe Institute of Technology (KIT), Hermann-von-Helmholtz-Platz 1, Eggenstein-Leopoldshafen, Karlsruhe 76344, Germany; 2 Department of Chemistry, University of Cambridge, Lensfield Road, Cambridge CB2 1EW, UK; 3 Department of Physics, NanoPhotonics Group, Kapitza Building, Cavendish Laboratory, University of Cambridge, Cambridge CB3 0HE, UK

**Keywords:** aluminum plasmonics, plasmonic metasurfaces, polymer blends, self-assembly, SERS, structural color

## Abstract

Subwavelength metal-dielectric plasmonic metasurfaces enable light management beyond the diffraction limit. However, a cost-effective and reliable fabrication method for such structures remains a major challenge hindering their full exploitation. Here, we propose a simple yet powerful manufacturing route for plasmonic metasurfaces based on a bottom-up approach. The fabricated metasurfaces consist of a dense distribution of randomly oriented nanoscale scatterers composed of aluminum (Al) nanohole-disk pairs, which exhibit angle-independent scattering that is tunable across the entire visible spectrum. The macroscopic response of the metasurfaces is controlled via the properties of an isolated Al nanohole-disk pair at the nanoscale. In addition, the optical field confinement at the scatterers and their random distribution of sizes result in a strongly enhanced Raman signal that enables broadly tunable excitation using a single substrate. This unique combination of a reliable and lithography-free methodology with the use of aluminum permits the exploitation of the full potential of random plasmonic metasurfaces for diagnostics and coloration.

## Introduction

The field of plasmonics has been highly dominated by gold (Au) and silver (Ag) for decades, mainly because of their low ohmic losses, robustness to oxidation, and strong optical dipolar interaction. However, comparable performances can also be achieved using aluminum (Al), which was the very first material in which the phenomenon of surface plasmons was demonstrated^1^. Recently, the plasmonic community has begun to look for alternative plasmonic materials^2, 3^, and the natural abundance of Al, its amenability to integration and its optical response throughout a broad wavelength regime from the ultraviolet (UV) to the near-infrared (NIR)^4, 5, 6, 7, 8, 9, 10^ make it attractive for the large-scale fabrication of nanostructures.

However, in the case of subwavelength-nanostructured metal-insulator metasurfaces that control light on length scales beyond the diffraction limit, reliable manufacturing is constrained not only by the material choice but also by expensive top-down lithographic techniques^11, 12, 13, 14, 15^. To date, this has hindered the economic affordability of plasmonic metasurfaces. However, such metasurfaces have been demonstrated for several interesting applications, such as structural coloration^6, 7, 14, 15, 16, 17, 18^ and sensing^19, 20, 21, 22, 23^. Their angle-independent scattering property makes plasmonic metasurfaces suitable for structural color production, which allows avoiding the use of toxic colorants. At the same time, strong field confinement is achieved at the scattering elements, which can be exploited to enhance Raman scattering (SERS) from molecules near the surface.

Here, we demonstrate a truly scalable fabrication route for producing plasmonic metasurfaces using only the spin coating of a binary polymer blend solution and metal evaporation. A metasurface produced in this way consists of a dense and random distribution of Al nanohole-disk pairs. The near-field coupling of localized surface plasmons (LSPs) at individual nanodisks and nanoholes controls the far-field scattering response. We demonstrate that these engineered surfaces are suitable for application such as structural color with an angle-independent appearance and surface-enhanced Raman spectroscopy (SERS) with uniquely strong, uniform, broadband optical-field confinement.

## Materials and methods

### Fabrication of metasurfaces

As the first step in the fabrication process, glass substrates were sequentially cleaned with acetone, isopropanol, and distilled water and then dried with nitrogen. Poly(methyl methacrylate) (PMMA, *M*_w_=5.09 kg mol^−1^, Polymer Standards Service GmbH, Mainz, Germany) and polystyrene (PS, *M*_w_=3.25 kg mol^−1^, Polymer Standards Service GmbH) were dissolved in methyl ethyl ketone (MEK, Sigma-Aldrich Co. LLC, St Louis, MO, USA) at mass ratios of 7.5:2.5, 7:3 and 6.5:3.5 to produce three solutions for the fabrication of blue, green and red metasurfaces, respectively. The concentration of the solutions was held fixed at 20 mg ml^−1^. The solutions were spin coated onto the glass substrates at a speed of 3500 r.p.m. and an acceleration of 2000 r.p.m. s^−1^ for 30 s. The relative humidity was maintained between 40% and 50% during spin coating. The samples were then rinsed twice in cyclohexane for 60 s and dried in a stream of N_2_ to remove the PS islands. Afterward, aluminum (UNIVEX 450, Oerlikon Leybold Vacuum GmbH, Köln, Germany) was evaporated onto the samples; the thickness of the deposited metal was monitored using a quartz crystal microbalance.

### Topographic analysis

The surface patterns were examined using scanning electron microscopy (SUPRA 60 VP, Carl Zeiss NTS GmbH, Oberkochen, Germany) and atomic force microscopy (Dimension Icon, Bruker Corporation, Germany branch, Karlsruhe, Germany). ImageJ (http://imagej.nih.gov/ij/) was used to perform a statistical analysis of the fabricated samples. The scanning electron microscope (SEM) and atomic force microscopy (AFM) images were converted into binary images, and the built-in ‘Analyze’ plugin was applied to perform a histogram analysis of the diameters. The two-dimensional Fourier power spectrum was obtained from the AFM data using the NanoScope Analysis software package (Bruker Corporation).

### Optical analysis

A customized optical microscope operating in dark-field (DF) mode was used for the micro-spectroscopic investigation of the fabricated samples. A halogen lamp was used as a light source using a 100 × objective (EC Epiplan-APOCHROMAT, Zeiss) with a numerical aperture of numerical aperture (NA)=0.95. The scattered light was collected in a confocal configuration using a 50-μm core optical fiber (Avantes, Leatherhead, Surrey, UK) and analyzed using a spectrometer (AvaSpec-HS2048, Avantes). The spatial resolution of the collected spectra was ~1 μm. Larger-area spectroscopic characterizations were performed using a 600-μm optical fiber, permitting a spatial resolution of ~25 μm. Furthermore, the angle-resolved scattering was measured using a home-built optical goniometric setup^24^. Light from a deuterium-halogen lamp (DH-2000, Ocean Optics, Dunedin, FL, USA) was collimated to form a 1-mm-wide parallel incident beam that illuminated the sample at a fixed angle. The scattered light was detected at multiple angles with an angular resolution of 1° and coupled into an optical fiber connected to the spectrometer (QE65000 Ocean Optics). All spectra were referenced to a white Lambertian reflectance standard (Spectralon, ≈99% reflectance).

### FEM simulations

Three-dimensional (3D) modeling of the coupled nanohole-disk system was performed using the finite element method (FEM, COMSOL Multiphysics, Göttingen, Germany) based on the model tutorial ‘Scatterer on a Substrate’. The unit cell was taken to be 200 nm and was surrounded on all sides by 200-nm-thick perfectly matched layers. The thickness of the PMMA was held fixed at 70 nm while both the nanohole-disk diameter and the Al thickness were varied. The refractive indices of the PMMA and the substrate were assumed to be *n*=1.5 (Ref. 25), and the wavelength-dependent optical indices of Al were extracted from the literature^26^. The scattering cross-section was calculated for a normally incident plane wave by integrating the scattered intensity (Poynting vector) over the closed surface of the nanohole-disk system and then normalized with respect to the incident intensity.

### SERS measurements

Biphenyl-4-thiol was dissolved in anhydrous ethanol to obtain a 1 mM solution^27^. Self-assembled monolayers were deposited by immersing an Al-coated substrate in the 1 mM solution for 20 h. Afterward, the sample was thoroughly rinsed with ethanol to remove unbound excess thiol and blown dry using nitrogen. SERS measurements were performed using a Renishaw inVia Raman microscope. The samples were illuminated with four different excitation lasers (488, 532, 633 and 785 nm) using a 50 × microscope objective (NA 0.75).

## Results and discussion

**Figure 1:**
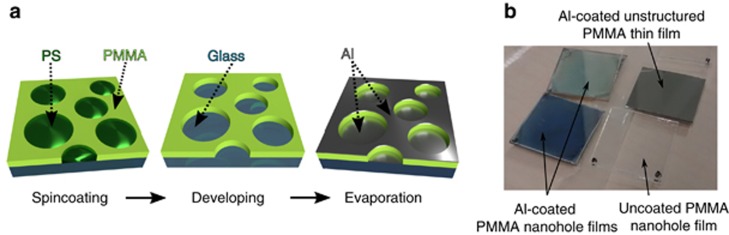
Fabrication of plasmonic metasurfaces by means of polymer blend lithography. (**a**) The fabrication process for a plasmonic metasurface consists of three steps: the spin coating of the PMMA-PS mixture, the developing of the PS with cyclohexane and the evaporation of the Al. The resulting plasmonic metasurface contains a dense distribution of nanohole-disk pairs. (**b**) A visual comparison of structured and non-structured surfaces with and without metal coatings. A spin-coated layer of PMMA with holes on glass appear transparent, whereas a 70-nm-thick unstructured PMMA film coated with 40 nm of Al exhibits the typical reflective appearance of aluminum. By combining the nanohole matrix with a metal coating, the surface properties can be tuned to yield on-demand coloration. As examples, blue and green samples are shown here.

The plasmonic metasurfaces were fabricated using a three-step procedure, as illustrated in Figure 1a. The first step consisted of an implementation of the bottom-up method known as polymer blend lithography^28, 29^. In brief, by controlling the spin-coating process of two phase-separated polymers under specific humidity conditions, we obtained a self-assembled nanostructured film consisting of a circular inclusion of the minor-phase polymer surrounded by the major phase. In detail, we spin-coated PMMA and PS in a blend solution of MEK onto a glass substrate. Because of the difference in polarity between PMMA and PS as well as the different evaporation rates of water and MEK, the PS/PMMA/MEK system undergoes a 3D phase separation when spin cast in a humid environment. Rapid precipitation of the PS molecules during spin coating leads to the self-assembly of nano-islands in the PMMA matrix. In the second step, we chemically etched the PS islands with cyclohexane to form a nanohole pattern in the PMMA matrix, and finally, we directionally evaporated a thin aluminum layer on top of this substrate to form nanohole-disk pairs. The characteristics of the individual nanohole-disk pairs determine the optical appearance of the resulting surface, which displays a uniform coloration, as shown in Figure 1b. Such an angle-independent structural color phenomenon is typical of plasmonic nanostructures and allows high color purity to be achieved using only ultrathin layers of metal^6, 7, 16, 18, 30^. This is in contrast to dielectric biomimetic structures, in which the color change as a function of the angle is prominent even for large thicknesses^31, 32^.

The macroscopic angular response of the surfaces was characterized using spectroscopic goniometry. Similar to most other plasmonic systems^6, 10, 18^, the fabricated metasurfaces exhibit angle-independent scattering properties (Supplementary Fig. S1), which make them interesting for structural color applications with high color purity. Even for a large angle of incidence of 80°, we observed only a spectral shift of 30 nm, which does not significantly affect the macroscopic coloration of a sample.

**Figure 2:**
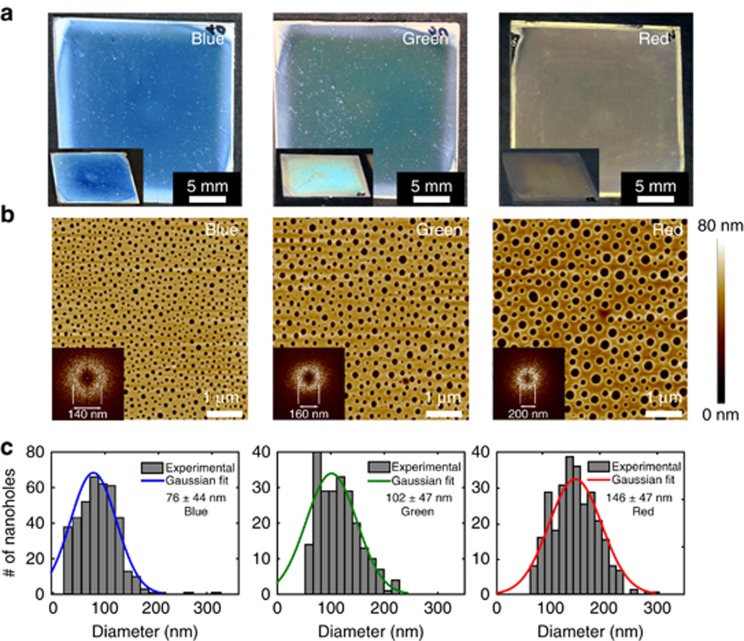
Surface topographies of PMMA samples with different diameter distributions. (**a**) Top views of the three samples show blue, green and red colorations. The oblique views presented in the insets confirm the angle-independent structural coloration of the metasurfaces. (**b**) AFM images of the blue, green and red samples reveal the height of the PMMA matrix. The corresponding two-dimensional power spectral densities of the AFM images shown in the inset reveal random nanohole distributions with average distances of 140, 160 and 200 nm. (**c**) A statistical analysis of the nanohole diameters determined from the AFM images. All three histograms were fitted with a normal distribution to find the mean diameter of the nanohole distribution for each sample.

The color appearance of the metasurface can be tailored by varying the average dimensions of the scatterers and their density (Figure 2a and 2b). The sizes of the holes as measured via AFM follow a Gaussian distribution. As shown in Figure 2c, the fabricated blue, green and red metasurfaces have hole-size distributions with maxima centered at 76, 102 and 146 nm, respectively. The back-scattered reflection is very intense because the nanohole-disk pairs are densely packed. Depending on the color, the average distance between the nanohole-disk pairs is between 140 and 200 nm, as measured directly from the fast Fourier transforms of the corresponding AFM images (provided in the inset of Figure 2b). Such a hole distribution can be tailored by adjusting the mass ratio between PS and PMMA, the molecular chain length, and the spin-coating parameters (solvent concentration and ambient humidity)^29^. In the following discussion, we report only results obtained by varying the mass ratio of PS and PMMA for a fixed molecular chain length. The perforated PMMA matrix is transparent (Figure 1b) and has a thickness of 70 nm, whereas the thickness of the Al layer is 40 nm. For comparison, we show in Figure 1b that aluminum coatings of the same thickness deposited on unstructured PMMA exhibit the typical reflective properties of Al metal.

**Figure 3:**
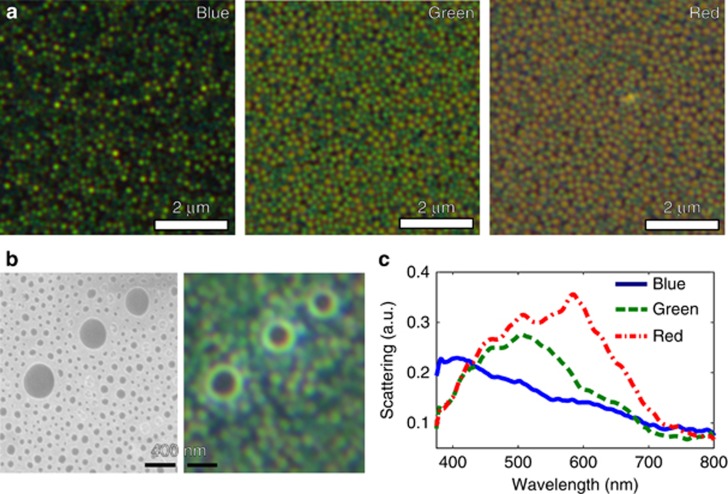
Dark-field measurements of the random plasmonic metasurfaces. (**a**) Dark-field images of blue, green and red samples, respectively. Each sample shows a distribution of colored spots because of the normal size distribution of the nanohole-disk pairs. (**b**) A one-to-one comparison of SEM and DF images of a green sample reveals the nanoscale origins of the scattering from individual nanohole-disk pairs, which is unperturbed by neighboring pairs. (**c**) The DF spectra of the samples show resonances in the blue, green and red, corresponding to their visual appearances.

The spectral response of each metasurface as characterized in the DF configuration is displayed in Figure 3. Scattering from individual nanohole-disk pairs appears in the DF images as a pixelated pattern (Figure 3a). By imaging the same area of the sample using an optical microscope and a SEM, we were able to correlate the position of each nanohole-disk pair with the presence of a bright-colored spot (Figure 3b). In particular, the macroscopically blue samples were found to produce blue and green spots. Similarly, the green samples mainly yielded green and orange spots, whereas for the red samples, orange and red spots were identified. The DF scattering spectra of the three samples are shown in Figure 3c. The scattering signal was measured in a confocal configuration to make it possible to ensure that the collected light had been predominantly scattered from a single spot. Three different resonances correlated with the average nanohole-disk dimensions were observed at 420 nm (blue), 510 nm (green) and 600 nm (red). No substantial difference was found in the scattering signal when the measurements were repeated 6 months after the fabrication of the samples, demonstrating the stability and robustness of the fabricated metasurfaces. This stability probably originates from the native self-limiting thin Al oxide layer, which prevents changes in the oxidation state of the aluminum^6, 8, 10^. This native oxide layer (≈3 nm) introduces a very small red shift in the scattering, but it does not significantly influence the overall optical behavior (Supplementary Fig. S2).

**Figure 4:**
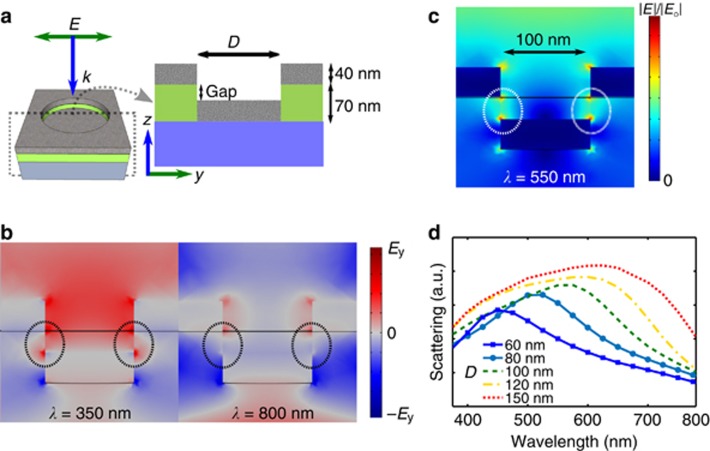
Three-dimensional simulations of a single nanohole-disk pair. (**a**) Cross section of the simulation model. The thickness of the PMMA layer used in the calculation is 70 nm, whereas the thickness of the Al layer is 40 nm. (**b**) The electric field distributions at short (350 nm) and long (800 nm) wavelengths reveal the ‘in-phase’ and ‘out-of-phase’ coupling, respectively, of the charge distributions. (**c**) The normalized electric field for the peak scattering wavelength (550 nm for *D*=100 nm) reveals the coupling between the dipolar resonances of the nanohole and nanodisk. (**d**) The calculated scattering spectra for different nanohole-disk diameters (keeping all other parameters constant) demonstrate the tunability of the dipolar resonance of a coupled nanohole-disk pair. The resonance becomes broader and more red shifted as the nanohole-disk diameter increases from 60 to 150 nm.

To better characterize the scattering response from a single nanohole-disk pair, we conducted simulations using the 3D FEM. A schematic view of the modeled structure is depicted in Figure 4a. The scattering from a nanohole-disk pair is the result of a strong dipole-dipole coupling between the plasmonic resonance of the individual nanohole and that of the nanodisk above it, as is visible in the electric field plot (Figure 4b and 4c). Such an interaction can be described by the hybridization theory for two adjacent plasmonic nanostructures^6, 10, 33^. The electric field profiles (Figure 4b) indicate the so-called ‘bonding’ (800 nm) and ‘anti-bonding’ (350 nm) modes of the coupled geometry. The in-phase component of the charge distributions of the top nanohole and bottom nanodisk corresponds to the anti-bonding mode, whereas the out-of-phase charge oscillations correspond to the bonding mode (Figure 4b). The coupling strength strongly depends on the distance between the nanohole and nanodisk, which are separated by PMMA and the thickness of the evaporated Al^30, 34^. The wavelength response as a function of the size of the nanohole-disk pair is found to be dominated by the localized plasmonic resonance of the nanodisk. However, the mode hybridization allows the plasmonic resonance to be efficiently coupled to the out-of-plane far-field excitation.

Experimentally, we observed that the diameters of the nanohole-disk pairs determine the color appearance of the metasurface: a larger diameter corresponds to a more red-shifted resonance (Figure 3c). Such spectral behavior is well confirmed by our simulations (Figure 4d), in which the pixel diameter was varied while keeping the thicknesses of PMMA and Al fixed. The different nanohole-disk pairs in the metasurface independently contribute to its macroscopic appearance (Figure 3a), which is the result of the averaged response of every nanoscale colored spot from each individual nanohole-disk pair. Despite the Gaussian distribution of pixel colors, the back-scattered wavelength largely depends on the mean of the dense Gaussian pixel distribution. However, because of the broad scattering from large nanohole-disk pairs, the color vibrancy of the red samples is not as great as that of the blue and green samples, which causes the red samples to appear slightly brownish (Figure 2a). Previous studies that have achieved variable scattering using nanohole-disk systems^7, 14, 16, 34^ have mostly considered strictly periodic structural designs (in both simulations and experiments). However, our work demonstrates that a controlled macroscopic scattered color can be achieved with individual nanohole-disk scatterers, even for a random distribution of individual pixels. This is particularly interesting because it reduces the need for strictly controlled metasurface fabrication methods, facilitating the development of cost-effective large-scale self-assembly techniques.

Moreover, the random distribution of the nanohole-disk pairs is responsible for the angle-independent structural color observed in the goniometric scattering measurements (Supplementary Fig. S1). Two reasons can be identified for this behavior. First, an aperiodic pixel distribution with an isotropic Fourier space distribution (Figure 2b, inset) prevents a directional scattering response on the macroscopic level^35^. Second, the average subwavelength distance between nanohole-disk pairs is invariant with respect to the angle-dependent surface plasmon polariton (SPP) modes that are present at metal-dielectric interfaces^6^. Therefore, aluminum metasurfaces do not show angle-dependent scattering if no interaction between the SPP and LSP modes is assumed at large angles.

**Figure 5:**
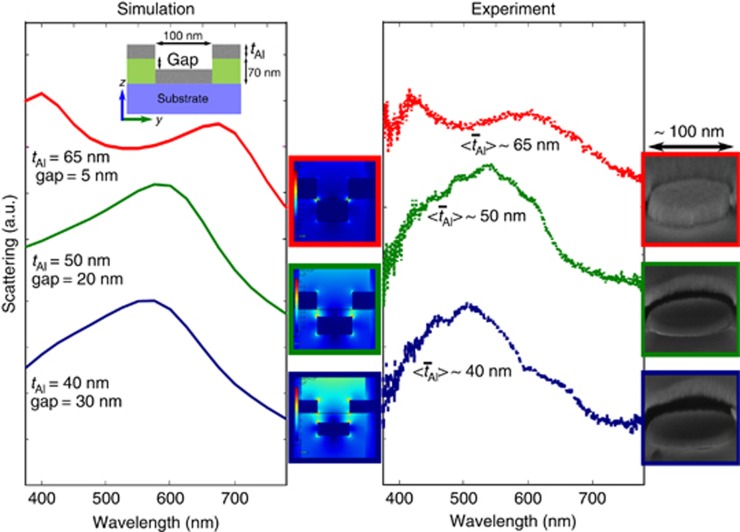
Simulated and experimental coupling effect. The calculated spectra for different nanohole-disk geometries, obtained by varying the thickness of the Al while keeping the PMMA thickness and the hole diameter fixed to 70 and 100 nm, respectively, are shown on the left side. The enhanced coupling of the optical fields within the nanocavities is clearly observed in the electric field norm distribution at the peak scattering wavelength in the near-field (shown in the middle) for larger Al thicknesses (smaller gaps between nanohole-disk pairs). The experimental DF spectra and SEM images of the corresponding geometries are shown in the right panel, demonstrating good agreement between the simulated and experimental results.

To inform the tuning of the coupling strength in the nanohole-disk pairs, we studied their resonance behavior as a function of the Al-layer thickness for a fixed PMMA thickness (70 nm). The calculated spectra indicate a red shift of the dipolar scattering peak with increasing Al thickness (Figure 5, left). An increase in the thickness of the Al layer results in a smaller gap between the individual plasmon resonances of the hole and disk, resulting in a larger coupling strength, which leads to a reduction in the resonance frequency of the system (that is, an increase in the resonance wavelength)^34, 36^. In the case of very small gaps (<10 nm), high-order (quadrupolar) resonances contribute in the blue spectral region. Such high-order resonances are generally weakly coupled to photons, and their scattering efficiency is smaller compared with the main dipolar resonance^37^.

However, an enhanced scattering efficiency of the quadrupolar mode for a 5-nm gap (70 nm PMMA matrix, 65 nm Al) might originate from the plasmonic coupling of high-order resonances. To compare the calculated scattering cross section of the single nanohole-disk configuration with the experimentally measured scattering response for various Al thicknesses, the Al-coating thickness was varied from 40 to 65 nm with a fixed 70 nm thickness of the PMMA matrix in both experiments and simulations. In this way, the gap height was varied between 5 and 30 nm. The corresponding SEM images are provided as insets in Figure 5. The average pixel diameter was chosen to remain fixed at 100 nm, and individual pixels were characterized. The presence of a gap between the disks and the upper layer of our system was revealed using a lift-off technique. The PMMA matrix with the top Al nanohole surface was removed, and densely packed Al nanodisks remained on the substrate (Supplementary Fig. S3).

A comparison of the DF scattering response with the simulated results (Figure 5) confirms the predicted red shift of the scattering resonance and the emergence of a secondary peak at a lower wavelength (corresponding to the high-order plasmonic resonances). This behavior is accompanied by an increase in the coupling strength of the nanohole-disk system and, consequently, an increased localization of the light in the near-field regime. Therefore, the large field enhancement and the broadening of the overall scattering of the metasurfaces make them ideal candidates for SERS with broadband excitation^19, 20, 21, 22, 23^.

**Figure 6:**
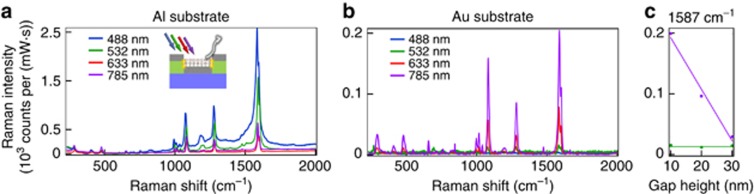
SERS analysis of the plasmonic metasurfaces. The surface-enhanced Raman scattering of BPT was measured using different excitation lasers for samples with a 70-nm PMMA matrix and (**a**) a 60-nm Al coating or (**b**) a 60-nm Au coating. (**c**) The Raman scattering intensity at 1587 cm^−1^ as a function of the gap height between nanohole-disk pairs, that is, the Au-coating thickness, for a 70-nm PMMA matrix under excitation at 785 and 532 nm.

To probe the SERS signals of self-assembled monolayers of biphenyl-4-thiol (BPT), we tested different metal-coated metasurfaces (PMMA 70 nm, Al and Au coatings, average nanohole-disk diameter of 100 nm) using different excitation lasers (488, 532, 633 and 785 nm). The obtained vibrational spectra of BPT (Figure 6a and 6b) are consistent with what has previously been observed in the literature^38^. For the Al-coated metasurface (Figure 6a), the Raman signal is strongly enhanced for all excitation wavelengths, following the broad plasmonic resonance spectrum measured via DF scattering spectroscopy (Figure 5). Notably, not only does the polydispersity of the nanoholes allow broadband enhancement, but individual nanoholes with 5-nm gaps also scatter broadband light because of the emergence of a quadrupole mode. (See Supplementary Fig. S4 for the calculated broadband enhanced optical field coupling.) The largest signal is obtained for excitation at 488 nm because of the plasmonic field enhancement introduced by the Al-coated SERS substrate and the spectral proximity of the excitation laser to electronic transitions in BPT (see ‘SERS enhancement calculations’ in Supplementary Information). By contrast, Au coatings produce the strongest signal under 785 nm excitation because of the red-shifted resonance of the material (Figure 6b). Excitation at 488 nm does not yield a signal because of the absorption characteristics of Au. The generally broadband resonant scattering is a result of the large spectral distribution of the pixel resonances. The enhancement factors (EFs) of the Raman signals reach 10^7^ (see ‘SERS enhancement calculations’ in Supplementary Information for details on the calculation). AFM measurements confirmed the monolayer coverage of the BPT on the Al and Au. The Raman enhancement strongly depends on the thickness of the metal coating. When the metasurface is excited at the plasmon resonance (785 nm for an Au coating), an increasing Au thickness results in a stronger Raman signal (Figure 6c). This increase is explained by the reduction in the gap size of the nanohole-disk pairs, leading to stronger near-field coupling of the system.

Most commercial SERS substrates are made from Au/Ag and exhibit typical effective Raman enhancements of 10^7^ or even less (Klarite: ∼10^6^)^22, 23^. Although Al has a smaller Q-factor than Au/Ag because of its large ohmic losses and the large radiative damping of plasmons in the visible regime, we achieve similar or even better enhancements using our geometry. The achievement of such an enhancement with Al might advocate for the replacement of existing Au/Ag SERS materials with Al, because doing so would drastically reduce costs and facilitate high scalability of Raman substrates, which are currently the issues hindering the commercial growth of SERS technology. In addition, current SERS substrates are mostly based on either rough metal surfaces or nanoparticles^16, 20, 22, 23^. Although a high surface roughness results in a high enhancement factor, controllability is a large concern. In this regard, our substrate is also random, but the enhancement can be precisely controlled by means of accurate metal deposition to create sub-10-nm gaps, thereby producing well-defined plasmonic ‘hotspots’. Nanoparticles, meanwhile, are often toxic (Ag), expensive (Au) and/or environmentally hazardous, and therefore, the interest of the community is shifting toward surface-based SERS substrates. Although most metal-insulator-metal (MIM) metasurfaces provide large electromagnetic enhancement and a platform for color generation^14, 15, 17, 18, 39^, their ‘hotspots’ are rarely accessible to analyte molecules. Our surface utilizes the same MIM concept but allows analytes to penetrate its plasmonic hotspots. Moreover, depending on the insulator material used, the selective physical adsorption of analytes to the insulator is possible in the case of our substrate. Therefore, analytes can selectively and directly access the hotspots, which is crucial for precise bio-sensing platforms.

Very recently, the self-assembly of metal nanostructures on insulator-metal stacks has begun to play an unprecedented role in the scalability of metasurfaces^20, 23, 39, 40^. For example, the thermal annealing^20, 23^ or colloidal synthesis and lithography^39, 40^ of metal nanoparticles have been utilized for light manipulation at subwavelength scales. However, the abovementioned techniques are restricted to a material choice of Au/Ag for visible light applications. Moreover, self-assembly via polymer phase separation is one of the most widely employed techniques for creating micro- and nanostructures for diverse applications. However, it is rarely used in the metasurface community because of the lack of dimensional controllability on length scales of 50–150 nm (ref. 41), which is required for the tuning of optical scattering by metals in the visible regime. Our technique is one of the first demonstrations of the direct fabrication of nanostructures via the self-assembly of laterally phase-separated binary polymers suitable for visible plasmonics applications.

## Conclusions

In conclusion, we successfully demonstrated a three-step procedure for fabricating robust and large-scale plasmonic metasurfaces based on a random distribution of plasmonic Al-based subwavelength nanostructures. The developed lithography-free method has several advantages over conventional lithography^42, 43^ because of its simplicity and scalability. The fabrication technique allows large-area metasurfaces to be obtained that show resonant responses in a wide range of wavelengths, thereby solving the issues related to the use of standard plasmonic materials such as Au and Ag^11, 12, 13^. The optical response of the fabricated metasurfaces can be tailored depending on the application. As an example, by varying the dimensions of the nano-scatterers, we can achieve metasurfaces with a high degree of color purity, which, in principle, can also be integrated with dynamic structural coloration techniques^44, 45^. Moreover, by varying the thickness of the deposited metal, we can achieve a broadband SERS response. This broad plasmonic response, extending from the UV to the NIR, originates from the dispersion of the nanohole diameters and the precise engineering of sub-10-nm nanogaps, which makes our surfaces unique compared with state-of-the-art SERS substrates^22, 23^. Finally, a large homogenous working area, that is, a large number of hotspots (billions per inch^2^ of area), permits simple and user-friendly handling, paving the way for the characterization of a large variety of biological molecules, such as proteins and DNA, which have electronic transitions in the UV^5^.
