# Supplementary material for: Scalable and controlled self-assembly of aluminum-based random plasmonic metasurfaces
Source: Light Sci Appl. 2017 Jul 14;6(7):e17015–. doi: 10.1038/lsa.2017.15 (PMC6062228; doi:10.1038/lsa.2017.15)
Supplement: Supplementary Information [file lsa201715x1.pdf]

Scalable and controlled self-assembly of aluminum-based random plasmonic metasurfaces

Supplementary Information

Radwanul Hasan Siddique[1, 2, #, *], Jan Mertens[3], Hendrik Hölscher[1], and Silvia Vignolini[2,*]

[1] Institute for Microstructure Technology, Karlsruhe Institute of Technology (KIT), Hermann-von-Helmholtz-Platz 1, 76344 Eggenstein-Leopoldshafen, Germany.

[2] Department of Chemistry, University of Cambridge, Lensfield Road, Cambridge CB2 1EW, United Kingdom.

[3] NanoPhotonics Group, Kapitza Building, Cavendish Laboratory, University of Cambridge, United Kingdom.

[#] Present address: California Institute of Technology (Caltech), Moore Laboratory, B127, MC 136-93, Pasadena, CA, USA.

**Corresponding Authors: rhs@caltech.edu, sv319@cam.ac.uk*

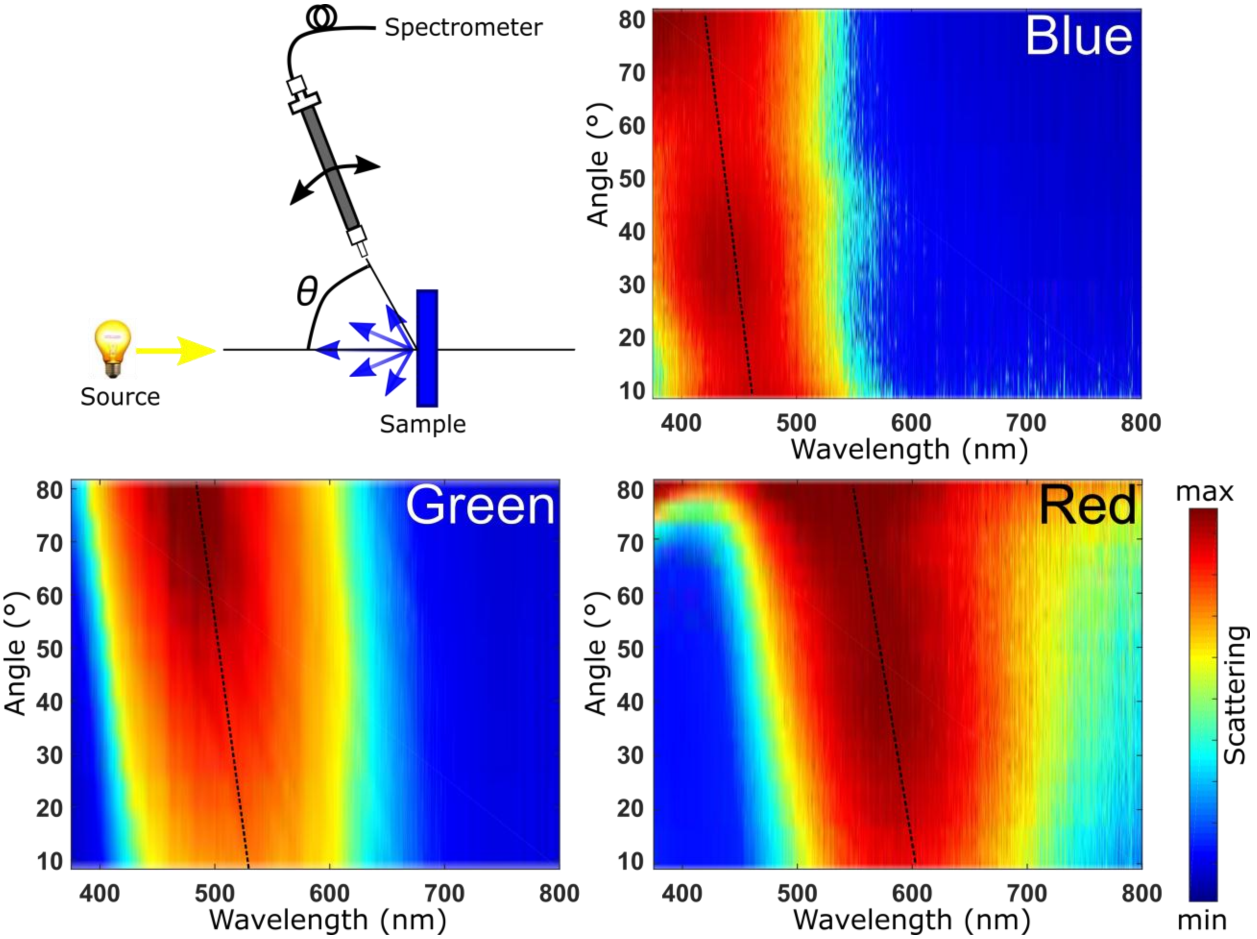

Figure S1 Angle-resolved goniometric scattering measurement. Angle-resolved goniometric scattering measurements of blue, green and red sample show the angle-independent scattering properties of the metasurfaces. The dotted lines show the shift of peak scattering wavelength.

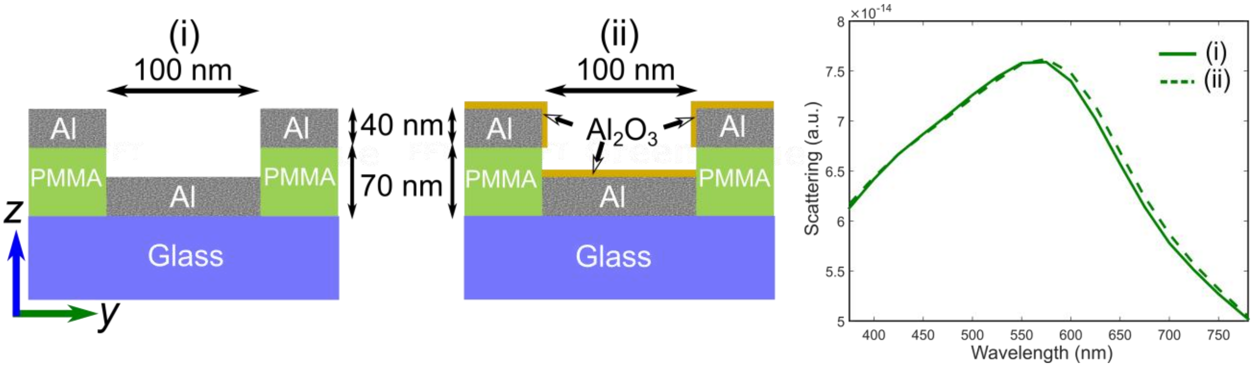

Figure S2 Effect of native aluminium oxide (Al_2O_3) on proposed random metasurfaces is shown with FEM simulation. There is a small red shift of 3 nm in the scattering behaviour observed due to the additional 3 nm insulating layer of Al_2O_3 (refractive index, n = 1.768 (ref. 1) is considered

for simulation). However, it does not significantly alter overall optical scattering.

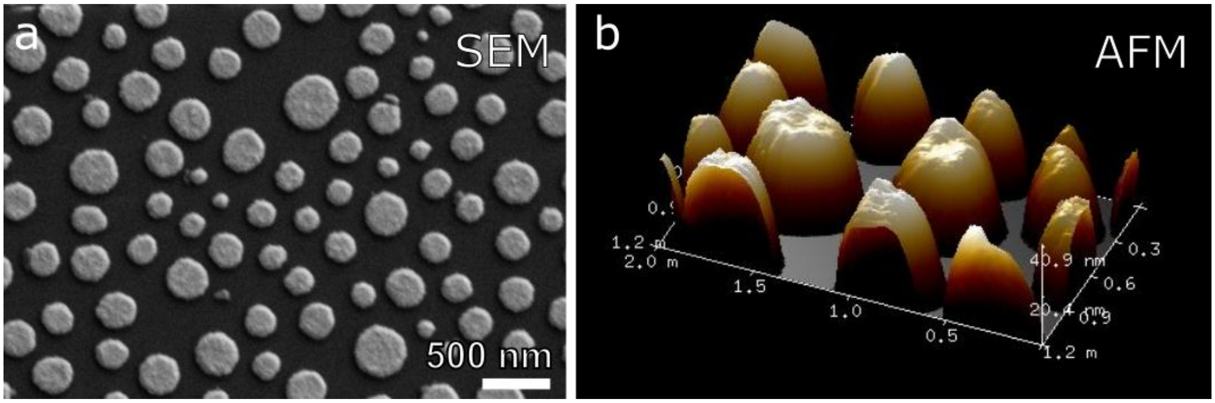

Figure S3 Plasmonic random metasurfaces lift-off. A lift-off technique is employed to prove the separation between the plasmonic elements by dissolving the PMMA layer, so the upper Al nanohole layer is removed and the Al disks keep remaining on the surface (a). (b) 3D surface topography confirms the height and homogeneity of the evaporated Al metal film.

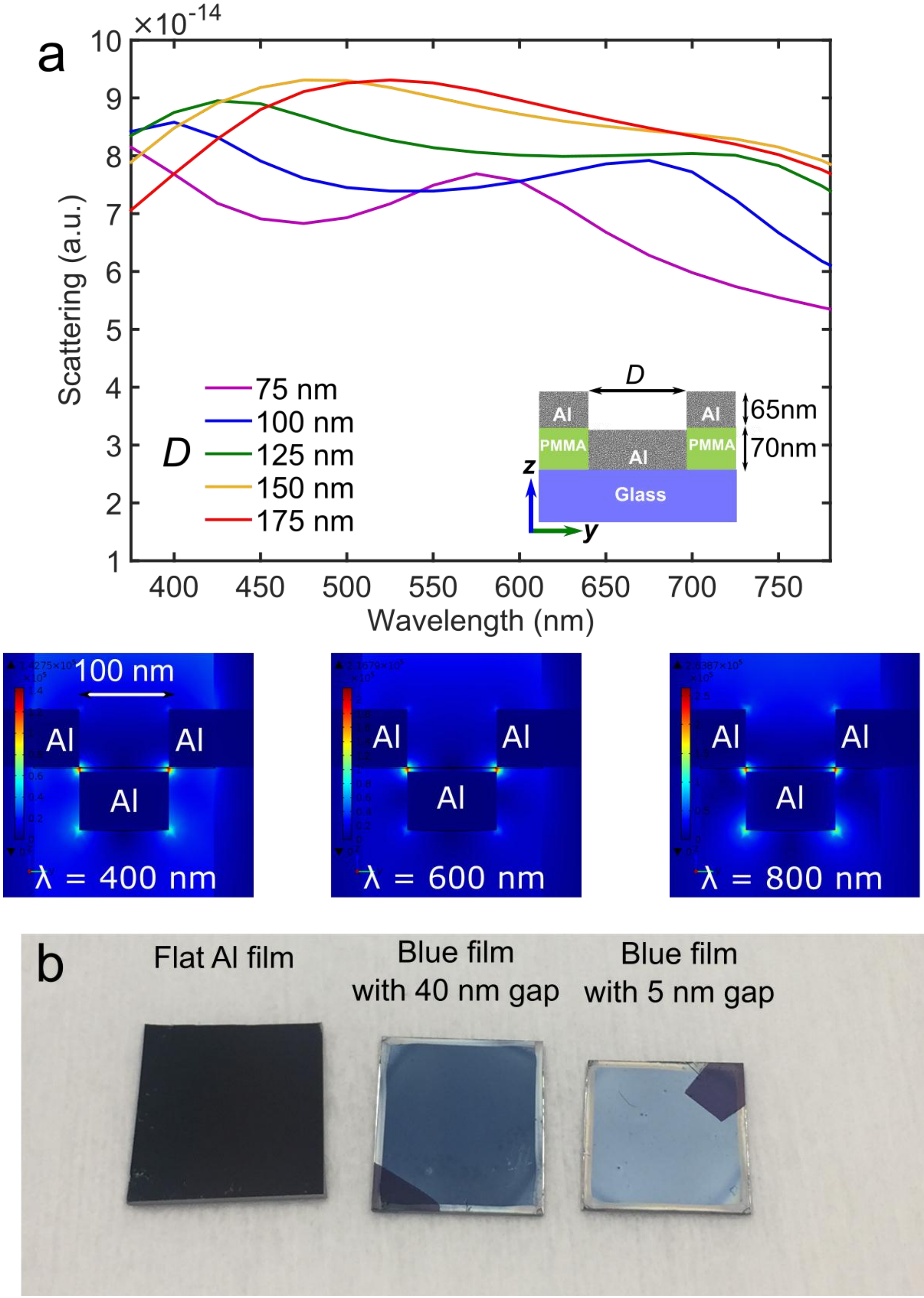

Figure S4 (a) 3D FEM Simulation of individual nanohole-disk is shown considering 5 nm gap for different diameters of hole. Enhanced coupling of optical fields within the nanocavities is observed in the electric field norm distribution at broad scattering peak wavelengths in the

near field leading to broadband scattering. The thickness of the PMMA layer used in the calculation is 70 nm, while the thickness of the Al layer is 65 nm to maintain a gap of 5 nm. (b) Photograph of a sample (right) with 5 nm gap between nanohole and nanodisk appears whitish compared to the one with a gap of 40 nm (middle). In both cases, average diameter of holes is maintained to be 80 nm, only the Al thickness is varied. For a comparison, a flat aluminium film is shown on the left.

SERS ENHANCEMENT CALCULATIONS

SERS enhancement factors (EFs) were calculated by EF = (I_{SERS}/N_{SERS}) / (I_{RAMAN}/N_{RAMAN}). First, the Raman scattering spectrum of 20 mM BPT in ethanol was measured. The signal strength was extracted at 1584 cm^{-1} and normalized to the mode volume following the $1/e^2$ method for Gaussian beams(ref.2), thus obtaining I_{RAMAN} and N_{RAMAN}. The numbers of molecules contributing to the SERS signal N_{SERS} was calculated by assuming a molecular surface coverage of 80 $Å^2$ per molecule(ref.3). The SERS intensity was extracted from the spectra shown in Figure 6a-b.

Approximated spot size: $d = 2\omega_0 = 2 \times \lambda/(\pi \times NA)$ and area of interest: $A=\pi \times \omega_0^2$.

Wavelength (nm)	Spot diameter (nm)	Area A $(\times 10^{-13} m^2)$
488	414	1.35
532	451	1.60
633	537	2.27
785	666	3.49

BPT density on substrate (ref. 4), $\rho_{BPT} = 1.25 \times 10^{18} \frac{\text{molecules}}{\text{m}^2}$

SERS signal measurements, $N_{SERS} = A \times \rho_{BPT}$:

Wavelength (nm)	$N_{SERS} \times 10^5$	Signal per molecule (Al)	Signal per molecule (Au)

		$I_{SERS}^{Al}/N_{SERS}^{Al}$	$I_{SERS}^{Au}/N_{SERS}^{Au}$
488	1.7	19.7 E-3	-
532	2	7.8 E-3	0.19 E-3
633	2.8	1.6 E-3	1.2 E-3
785	4.7	1.2 E-3	3.0 E-3

Raman signal measurements:

Sample	Wavelength (nm)	N_{RAMAN} (counts / (mW*s))
BPT on Al SERS substrate	488	3347
	532	1553
	633	450
	785	570
BPT on Au SERS substrate	488	-
	532	37
	633	332
	785	1433
BPT on flat Au	488	- (no signal)
	532	-
	633	-
	785	-

Calculated Raman enhancement, EF = $(I_{SERS}/N_{SERS})(I_{RAMAN}/N_{RAMAN})$:

Wavelength (nm)	EF ($\times 10^6$) (Al)	EF ($\times 10^6$) (Au)
488	1.5 (7.3)	-
532	2.6	0.6
633	3.4	2.5
785	15	38

REFERENCES

1 Zhu XL, Vannahme C, Højlund-Nielsen E, Mortensen NA, Kristensen A. Plasmonic colour laser printing. *Nat Nanotechnol* 2016; **11**: 325-329.

2 Periasamy A. *Methods in Cellular Imaging*. New York: Springer; 2013.

3 Azzam W, Wehner BI, Fischer RA, Terfort A, Wöll C. Bonding and orientation in self-assembled monolayers of oligophenyldithiols on Au substrates. *Langmuir* 2002; **18**: 7766-7769.

4 Cyganik P, Buck M, Azzam W, Wöll C. Self-Assembled Monolayers of ω-Biphenylalkanethiols on Au (111): influence of spacer chain on molecular packing. *The Journal of Physical Chemistry B* 2004; **108(16)**:4989-96.
